# Dirt Cheap and Without Prescription: How Susceptible are Young US Consumers to Purchasing Drugs From Rogue Internet Pharmacies?

**DOI:** 10.2196/jmir.1520

**Published:** 2010-04-26

**Authors:** Lana Ivanitskaya, Jodi Brookins-Fisher, Irene O´Boyle, Danielle Vibbert, Dmitry Erofeev, Lawrence Fulton

**Affiliations:** ^1^The Herbert H. and Grace A. Dow College of Health ProfessionsCentral Michigan UniversityMount Pleasant, MIUSA; ^2^School of Public HealthUniversity of MichiganAnn Arbor, MIUSA; ^3^Central Michigan UniversityMount Pleasant, MIUSA; ^4^McCoy College of Business AdministrationTexas State UniversitySan Marcos, TXUSA

**Keywords:** skill assessment,, health literacy, eHealth, health information skills, Internet pharmacies, counterfeit pharmaceuticals, cyberdrugs, cyberpharmacies

## Abstract

**Background:**

Websites of many rogue sellers of medications are accessible through links in email spam messages or via web search engines.  This study examined how well students enrolled in a U.S. higher education institution could identify clearly unsafe pharmacies.

**Objective:**

The aim is to estimate these health consumers´ vulnerability to fraud by illegitimate Internet pharmacies.

**Methods:**

Two Internet pharmacy websites, created specifically for this study, displayed multiple untrustworthy features modeled after five actual Internet drug sellers which the authors considered to be potentially dangerous to consumers.  The websites had none of the safe pharmacy signs and nearly all of the danger signs specified in the Food and Drug Administration´s (FDA´s) guide to consumers. Participants were told that a neighborhood pharmacy charged US$165 for a one-month supply of Beozine, a bogus drug to ensure no pre-existing knowledge. After checking its price at two Internet pharmacies—$37.99 in pharmacy A and $57.60 in pharmacy B—the respondents were asked to indicate if each seller was a good place to buy the drug. Responses came from 1,914 undergraduate students who completed an online eHealth literacy assessment in 2005-2008. Participation rate was 78%.

**Results:**

In response to "On a scale from 0-10, how good is this pharmacy as a place for buying Beozine?" many respondents gave favorable ratings. Specifically, 50% of students who reviewed pharmacy A and 37% of students who reviewed pharmacy B chose a rating above the scale midpoint. When explaining a low drug cost, these raters related it to low operation costs, ad revenue, pressure to lower costs due to comparison shopping, and/or high sales volume. Those who said that pharmacy A or B was "a very bad place" for purchasing the drug (25%), as defined by a score of 1 or less, related low drug cost to lack of regulation, low drug quality, and/or customer information sales. About 16% of students thought that people should be advised to buy cheaper drugs at pharmacies such as these but the majority (62%) suggested that people should be warned against buying drugs from such internet sellers. Over 22% of respondents would recommend pharmacy A to friends and family (10% pharmacy B). One-third of participants supplied online health information to others for decision-making purposes. After controlling for the effects of education, health major, and age, these respondents had significantly worse judgment of Internet pharmacies than those who did not act as information suppliers.

**Conclusions:**

At least a quarter of students, including those in health programs, cannot see multiple signs of danger displayed by rogue Internet pharmacies. Many more are likely to be misled by online sellers that use professional design, veil untrustworthy features, and mimic reputable websites. Online health information consumers would benefit from education initiatives that (1) communicate why it can be dangerous to buy medications online and that (2) develop their information evaluation skills. This study highlights the importance of regulating rogue Internet pharmacies and curbing the danger they pose to consumers.

## Introduction

In 2007, US adults spent out-of-pocket US $47.6 billion to buy pharmaceutical drugs and an additional US $14.8 billion out-of-pocket to purchase nonvitamin, nonmineral natural products [1]. Even in better economic times, some US patients could not afford pharmaceuticals and resorted to skipping medications, reducing doses, or leaving prescriptions unfilled [2]. A recent downturn in the US economy may have worsened cost-related medication nonadherence, especially among the poorest and the sickest.

Pressed to choose between buying expensive medications and spending on other basic needs, some health consumers go online to search for bargains. They find websites that boast low prices and advertise their readiness to dispense prescription drugs without a valid prescription. Because many of these websites are rogue, consumers are at risk for taking medications that are inappropriate for their health condition and that interact with other drugs they take. In addition, they may be sold unapproved, contaminated, impure, or fake drugs.

As conservatively estimated by the Center for Medicine in the Public Interest, the sales of counterfeit medicines will grow twice as fast as the sales of legitimate pharmaceuticals (13% as compared to 7.5%, annually, 2004 to 2010) [3]. The Internet is a global distribution channel for these fake medicines, but little is known about the extent to which consumers are able to buy medicines online safely. Are consumers evaluating pharmacy websites and paying attention to signs of low credibility, unsupported claims, and violations of privacy? If an illegitimate pharmacy offers prescription medications at a deep discount, how likely are consumers to buy these products? This exploratory study examined the ability of students enrolled in US higher education academic programs to determine the legitimacy of Internet pharmacies. If even college-educated individuals and those with specialized training in health-related sciences are enticed by low price tags and unsubstantiated claims offered by rogue online sellers of prescription drugs, then risks of purchasing drugs online could be even greater for America’s most vulnerable, such as less educated patients without prescription drug coverage whose failing health necessitates the use of multiple expensive drugs.

Creation of pharmacy websites coincided with the growth of the number of Internet users. Today, the majority of American adults are using the Internet. In 2008, 74% of adults were Internet users [4]. The rate of Internet use is even higher among younger, more educated individuals and those with higher incomes. Two of the most popular uses of the Internet are to find medical information and access health care research and findings. Although these numbers may be somewhat inflated due to social desirability bias [4], reports have suggested that 83% of American adults who use the Internet (or 61% of adults in the United States) seek health information online [5], and that many of these individuals rely on the Internet as their main source of health information [6]. Studies suggest that consumers use search engines to find health information but do not precisely specify their keywords or limit their searches in any way [7]. Only 15% of individuals seeking health information say they “always” check the sources and date, while an additional 10% stated they do so “most of the time [7].” This may indicate that 85 million Americans get health information without knowing the quality or legitimacy of the information provided [7].

With the increased commerce on the Internet comes increased risk for users. The average user accesses unregulated sites without the necessary skills to discern if these are trustworthy websites or dangerous ones [8]. Therefore, individuals learn about their health conditions from the Internet without knowing if the source is reputable or questionable. Internet users often underestimate the effort and competence required to review and search for trustworthy and credible health information. An uneducated search can lead to a greater risk of making health decisions on the basis of incomplete, out-of-date, or untrustworthy information, and the risk can exponentially increase for individuals with poor overall health literacy and poor eHealth literacy in particular [9].

While searching for health information online, consumers are offered advice about prescription medications, exposed to drug advertisements, and given links to websites that sell medications. Access, convenience, and privacy are potential benefits of Internet pharmacies for the consumer. Internet pharmacies increase access to drugs for those that are disabled or otherwise homebound. They also provide individuals with the convenience of 24-hour shopping, a huge selection of available drugs, and privacy for those who do not wish to discuss their medical conditions with pharmacists [10]. Some proponents of Internet pharmacies claim that paper prescriptions are often poorly written with illegible handwriting, wrong dosages, and inappropriate medications [10]. Proponents further claim that e-prescribing can often avoid these errors and save millions of dollars of health care costs [11].

There are also many concerns and risks associated with Internet pharmacies, most importantly, those related to using the Internet as a means of bypassing the usual regulatory systems [10]. In fact, Bessell and colleagues [12] found that even with tighter standards in many countries, consumers are still at risk for problems when buying nonprescription drugs from Internet pharmacies since balanced information about the medications may not be presented. Those who shop in Internet pharmacies—virtual patients—never meet the doctors or pharmacists who distribute their medications. A buyer can go to an Internet pharmacy online, select a particular prescription, and fill out a questionnaire. This questionnaire might be sent to a physician for approval, but this is not always required. As Besell and others found [12], drug interactions were not detected by the majority of e-pharmacy staff. The prescription is often filled in a location that is completely different from the location of the Internet pharmacy [13].

Many individuals may not have the ability to know what they are getting when they buy drugs online. Consumers are potentially receiving more counterfeit drugs due to large Internet sales (projected at US $75 billion by 2010) [14]. Internet pharmacies can also be seen as a last resort for individuals who are desperate for a cure to serious medical conditions and may be particularly susceptible to false claims [10]. Electronic records of dispensed medications, such as a national register or a personal record, will not be complete unless they include seller information that can be checked to identify rogue pharmacies [15].

Another major issue with Internet pharmacies is the potential for the buyer to purchase illegal substances. In addition to many legitimate Internet pharmacies that prescribe in accordance with local and federal laws, a great number of online operations offer controlled substances without regard for the prevailing national law [9]. In the United States, psychoactive drugs rank second only to marijuana as drugs of abuse, if tobacco and alcohol are discounted [9], while amphetamine-type stimulants are the second most widely used drugs in the world [16]. The Internet plays a significant role in global misuse of these stimulants, permitting uncontrolled dispensing by online pharmacies and providing information on techniques for illicit manufacture [16].

Although the US government has developed regulations and policies to protect its consumers from illegitimate Internet pharmacies, there are many implementation challenges. The biggest challenge stems from trying to regulate US pharmacies that are in offshore locations [17]. Another challenge is the current license status of the prescribing physician in a state other than where the patient receiving the prescription drug resides [17]. Additionally, in those online pharmacies where no physician is involved, patients cease to be patients and instead become consumers able to buy prescription medications (and possibly controlled substances) from anonymous providers offering no ongoing treatment relationship or responsibility for the drugs dispensed [8]. In these situations, regulatory concerns and the patient’s health and safety are not often the priority. If complications do arise from these medications, however, individuals return to the traditional medical systems to manage overdoses, addictions, and adverse drug effects and interactions [8] with providers that do not have adequate knowledge of the patient’s condition or status.

There are federal efforts underway to protect American citizens who utilize online pharmacies. According to the Food and Drug Administration (FDA), the distribution of controlled substances or dangerous pharmaceuticals without a valid prescription is illegal, and officials have had concerns about the safety of obtaining prescription drugs over the Internet for many years [10]. Their concerns center on the many individuals who may not have the ability to recognize that their purchases may be fraudulent. The FDA warns that drugs purchased over the Internet may be counterfeit or contaminated, the wrong drug, outdated drugs, or incorrect dosages, not to mention the possible ill effects of impure or unknown ingredients found in drugs manufactured in substandard conditions [18]. Web-based prescription monitoring programs help curb drug abuse and are spreading across the US. These programs aim to stop patients from doctor shopping, prescription forgery, and reckless prescribing of controlled substances [11]. At least 33 states have enacted Prescription Drug Monitoring Programs, and many others are considering them [11]. These programs have not been extended to all Internet pharmacies, especially those that are based outside of the US.

Additionally, the FDA encourages that prescription drugs and treatment regimens should be made with the advice of licensed health care providers who have access to the patient’s current health status and medical history. Under many of the recent laws, patients must be physically examined by a licensed health care practitioner the first time drugs are prescribed to determine if the drug is appropriate for treatment [10]. When the patient is using an Internet pharmacy, the health care provider is often not involved and cannot perform a physical examination. Therefore, the patient is self-diagnosing. This process also allows a consumer to misrepresent their medical information. Self-diagnosing, information misrepresentation, and lack of involvement of providers have implications for the medical system and doctor-patient relationship. The added burdens are inappropriate self-treatment, use of counterfeit or inaccurately labeled drugs, and adverse interactions with other medications, all of which may delay or complicate proper treatment. Doctor-patient relationship may suffer when patients request inappropriate treatments and misinterpret denials as cost cutting [19]. Another reason for relationship deterioration is physicians’ dismissal of questions that patients ask after searching for health information online [20].

Under the federal Food, Drug and Cosmetic Act, the FDA has the legal authority to take action against the importation, sale, or distribution of adulterated or misbranded drugs; the importation, sale, or distribution of approved new drugs; illegal promotion of a drug; the sale or dispensing of a prescription drug without a valid prescription; and counterfeit drugs [10,17]. When the Internet is used for an illegal sale, the FDA, working with the Department of Justice, must establish the same elements of a case, develop the same charges, and take the same actions as it would if another medium, such as a storefront or a clinic, had been used. The FDA has investigated and referred cases for criminal prosecution and initiated civil enforcement actions against online drug sellers [10].

In July 1999, the FDA adopted and implemented the Internet Drug Sales Action Plan to expand and improve the activities of the agency in addressing unlawful sales of drugs over the Internet [10]. The plan includes engaging the public by informing them about safe ways to purchase pharmaceutical products over the Internet; verifying the legitimacy of Internet sites dispensing pharmaceuticals; cooperating internationally with foreign governments; and customizing and expanding enforcement activity by establishing priorities, improving data acquisition, and coordinating case assessment [17]. Since 2000, the FDA has issued numerous cyber letters to online sellers suspected in illegal drug trade and in “promoting dietary supplement products with claims to diagnose, mitigate, treat, cure, or prevent a specific disease or class of diseases [21].”

Additionally, the National Association of Boards of Pharmacy (NABP) has developed a Verified Internet Pharmacy Practice Sites accreditation program and a website to help consumers identify Internet pharmacies that are out of compliance with state and federal laws or do not meet patient safety and pharmacy practice standards (http://www.nabp.net). Still, Palumbo et al [14] have stated that Congress needs to be more involved in curbing illegitimate online pharmacies. At this time, the US government has limited control over foreign Internet pharmacies. The FDA efforts include requesting other foreign governments to take action against the seller of the product, asking US Customs and Border Protection to stop the imported drug at a US port of entry [10], or sending warning letters to online sellers [21].

International cooperation is underway to combat online sales of illegal and counterfeit medicines. Coordinated by INTERPOL and the World Health Organization’s (WHO) International Medical Products Anti-Counterfeiting Taskforce, an Internet monitoring operation called Pangea II, focused on key elements of online drug sellers’ businesses—the Internet service provider, payment, and delivery. This five-day operation (November 16-20, 2009) involved 24 countries and “revealed 751 websites engaged in illegal activity, including offering controlled or prescription only drugs, 72 of which have now been taken down [22].” The first operation Pangea took place in 2008. It lasted one day and involved 8 countries [23]. Global press coverage of both operations was used to raise consumer awareness about counterfeit medicines.

While it is useful to take down established websites by illegal pharmacies, the online sellers often employ direct-to-consumer advertisement strategies, such as email spam messages with Web links to ephemeral websites. These websites are hard to track due to their transient nature. Gernburd and Jadad studied health spam offers and found that about half of online sellers of health products deactivated their spam links within a week of message delivery and three-quarters deactivated them after one month [24]. The oversight and regulation of ephemeral “cybersellers” who market directly to consumers would require continuous monitoring of email traffic. That is an enormous challenge because most email traffic is spam and because in any given month between 10% and 30% of spam messages fall into the category of health-related spam (higher spikes are possible if rogue sellers see an opportunity to capitalize on a global health issue or piggyback on press coverage, as was the case with H1N1) [24-26].

Given the global nature of the Internet and the challenge of regulating activities that cross national borders, federal efforts may be insufficient to protect US residents who purchase drugs online. Consumer education is likely to play an important role. An example of consumer education is an FDA consumer update titled “The possible Dangers of Buying Medicines Over the Internet [27].” It instructs consumers to look for the following signs of trustworthy pharmacies: a US location, a pharmacy license by the state board of pharmacy, complete contact information (patients can talk to a licensed pharmacist), and a requirement of a prescription from a licensed health care provider for any prescription medicine. The FDA update also lists the following signs that help detect rogue, unsafe pharmacies: no phone contact with pharmacy staff, medicines that are priced much lower than the average market price, an illegal practice of requiring no prescription, and poor protection of consumers’ personal information.

This study was designed to gain understanding of how individuals evaluate the websites of two Internet pharmacies that were specifically designed to show many of the unsafe signs and no signs of trustworthiness, as specified by the FDA consumer education materials.

The purpose of the study was to examine health consumers’ vulnerability to fraud by rogue Internet pharmacies. Since little is known about consumers’ judgment of online pharmacy features, in particular those of illegitimate sellers of prescription medications, this exploratory study is based upon secondary data from a convenience sample—a large group of university students who completed the Research Readiness Self-Assessment (RRSA). A health version of RRSA, an online interactive application, was designed to help information seekers to become effective, independent users of health information from digital (electronic) sources [28]. The assessment was used to obtain objective measures of competencies related to finding and evaluating health information. The evaluation module of the assessment included several questions about online pharmacies. Specifically, the assessment takers were asked to review two pharmacy websites, designed specifically for the purposes of the assessment. The features of these websites were common to websites of illegitimate online pharmacies. Responses by about 2000 individuals who completed the assessment between September 2005 and March 2008 were used to examine the degree to which college-educated information seekers are able to determine the trustworthiness of online pharmacies. The outcomes of this study can provide important insights for policy makers, authorities involved in regulating pharmacy operations, and consumer educators.

## Methods

### Research Design

Since September 2005, a cross-sectional online assessment titled Research Readiness Self-Assessment, Health Version (RRSA-Health) was administered to students, most of whom were enrolled in introductory health courses at a large Midwestern university. The study was approved by an institutional review board (IRB). The interactive online assessment contained questions about Internet pharmacies specifically designed for this study that showed multiple signs of low credibility.

### Focus Population

The findings of this study can be generalized to a population of healthy young adults who are in their early 20s and enrolled in college programs. These individuals have the requisite computer skills related to using email, navigating websites, and conducting basic searchers in popular search engines. Individuals in this age group are among the most active users of the Internet, who are likely to do information searchers for themselves and others, for example, less computer literate family members.

### Procedures

#### RRSA and its Administration

The RRSA is an online assessment of eHealth literacy skills, specifically, those related to finding and evaluating health information from digital sources. It is a combination of an e-survey and an e-test with detailed performance feedback and suggested resources for skill improvement. To complete the RRSA, participants needed basic computer skills that are now acquired at the high school level. The purpose, development, and administration of the RRSA were described in an earlier study by Ivanitskaya et al [28]. Since that publication, RRSA-Health was expanded to include new questions that measured the evaluation of health information, such as questions about a medical doctor’s credentials and the credibility of two Internet pharmacies that advertise drug prescription services based on an online questionnaire rather than a physical exam by a doctor. To assess how students would evaluate these online pharmacies, six new items were developed, as well as seven additional items that asked students to explain low drug costs. The addition of new questions lengthened the average completion time from 26 to 37 minutes.

The link to an assessment was given via an email and posted on a course website. In addition, instructors who taught face-to-face courses advertised the RRSA in class. A password was required to register for and then to participate in the assessment. The participants were informed that their participation was voluntary, that the assessment takes about 35 minutes to complete, and that their aggregate data may be used for research purposes. The primary investigator’s email address was provided, and the purpose of the study was explained. Access to online respondent data was restricted through a password, an identification of a unique IP address, and a 60-minute time limit.

#### Development of Rogue Pharmacies and Measures

The two pharmacies featured in the assessment had a large number of untrustworthy features (see Table 1) and no signs of trustworthiness listed in the recent FDA update [18]. Students accessed the two websites by clicking on links provided in the RRSA questions. The pharmacy websites were kept on a local server. Their pages could be navigated by clicking on buttons labeled “home,” “contact,” “search,” “about us,” “FAQ,” and “disclaimer.”

**Table 1:** Features of online pharmacies used in this study

Feature	Pharmacy A (URL extension: .net)	Pharmacy B (URL extension: .com)
Advertising claims (as they appeared in the source)	“Beozine—US $37.99—now available in a gel!” “No prescription required! Our staff can prescribe medications based on a detailed questionnaire. We would review the information you submit and respond within one hour! Order prescription medications without leaving home! Low low prices!!!! Next-day delivery. World-wide delivery. Easy and secure ordering. FREE medical review with prescription from real doctor. We proudly serve customers who know how to find a good price.”	“Beozine retails for US $200, we sell for $59.50!” “Get medications without the hassle, embarrassment, and cost of the doctor's office and pharmacy. Everything is done online and confidentially. 1000s of low cost pharmaceuticals, wholesale pricing, prescription updates, worldwide shipping, private online ordering, and discreet packaging. No need to meet your doctor if your prescription expired. Discount generic drugs, save over 70%. Our competitors can’t match our prices! INTEGRITY IS TRULY EVERYTHING!!!!!”
Prescription process	Fill out and submit an online questionnaire. No prescription is required.	Submit a valid prescription by FAX or email (with a scanned prescription attached) or request an updated prescription.
Contact options	Pharmacy’s physical address (outside the US), online contact form, and email address.	Pharmacy’s physical address (outside the US), toll-free FAX, online contact form, and email address.
Information requested from customers	Name, date of birth, email address, mailing address, detailed insurance information, specific medical problems, all past surgeries, conditions treated with each surgery, all medications they plan to take, and all current medical conditions.	All over-the-counter and prescription medications they are currently taking, the length of time for each, and medications they plan to take in the near future.
Promises and disclaimers	“Any information provided by our customers is never shared, sold, or released to any third party outside of our network of doctors, who need to view the information in order to write and fill a prescription and our network of partners.” Customers must agree with a responsibility statement: “All questions asked of me during the medication request have been answered truthfully and completely.”	“By requesting this medication the requestor confirms the release of pharmacy and all of its employees and contractors, including doctors, from ANY and ALL liability whatsoever associated or connected with the request for and use of medication. The statements have not been evaluated by the FDA. No advice or product listed here is intended to diagnose, treat, cure, or prevent any disease.”
Statements to reassure customers	“Our organization is committed to meeting and exceeding current regulations. We utilize licensed doctors. Our pharmacies are licensed to ship medication worldwide and employ licensed pharmacists to provide you with the highest standards of pharmaceutical care.” “Online consultations are the latest concept in health care.”	“Rest assured you are receiving the same medication as you would at your neighborhood pharmacy.” “As a marketing group primarily involved in membership-based ordering service promotion, we established relationships with the largest pharmaceutical wholesalers. We don't sell any type of medications, we are here just to help members get cheap medications.”
Customer testimonials, examples	No testimonials.	“I tried your pharmacy after I read a testimony of a customer who got a new prescription in 15 minutes. I am so happy I did not have to go see an expensive doctor...”

Designed for the health version of the RRSA, the two pharmacies were closely patterned after five actual Internet pharmacies that the first author accessed in 2005 by searching for the phrase “no prescription required” in Yahoo and Google. Researchers who recently studied characteristics of Internet pharmacies reported that 96 of 118 drug sellers did not require a medical prescription [29]. The two websites were designed to show that the pharmacies were located outside of the US. Just like the original sellers, these pharmacies could be contacted by FAX, via email, or by submitting a comment typed into an online textbox. No phone numbers were given to contact a live person. Posted on their websites were misleading statements (“we don’t sell any type of medication, we are just here to help members get cheap medications”), suspicious disclaimers (“by requesting this medication the requestor confirms the release of pharmacy and all if its employees and contractors, including doctors, from ANY and ALL liability whatsoever associated or connected with the request for and use of medications”), and unsupported claims (“rest assured that you are receiving the same medication as you would at your neighborhood pharmacy”). Also of concern was the large amount of personal information requested from customers. As promised by pharmacy A, “any information provided by our customers is never shared, sold, or released to any third party outside of our network of doctors, who need to view the information in order to write and fill a prescription and our network of partners.” Although crafted as a reassuring statement, the undefined “network of partners” may include nearly anyone. Both websites requested consumers’ personal information and provided no phone number to contact their staff. Similar to the original websites on which the two pharmacies were modeled, the online text contained grammatical mistakes and typographical errors. Previous studies demonstrated that surface credibility, defined as attractive design or professional appearance, plays an important role in building consumer’s confidence in the website [30,31]. The two pharmacy websites used in this study were designed to display below average surface credibility. Therefore, it is unlikely that many study participants were impressed by the design or appearance of the websites.

Among the measured variables were students’ evaluations of the two pharmacies. The students were presented with a scenario: “You have been prescribed the drug Beozine. Your out-of-pocket cost at your neighborhood pharmacy is $165 for a one-month supply of this drug. While searching for cheaper options, you found two online pharmacies. Suppose you have a credit card and do not mind using it online. Click on the line to indicate how good pharmacy [name linked to pharmacy’s website] is as a place to buy a drug called Beozine, which costs $165 at your neighborhood pharmacy.” The students were then instructed to rate each pharmacy using an electronic visual analog scale (eVAS), designed as an online slider. The slider had 400 possible points located on a “click or drag” scale that ranged from 0 (very bad) to 10 (very good). As a proxy measure of their intent to use the two pharmacies, students were asked to agree or disagree with the following statements, “I would recommend [Pharmacy name] to my friends or family,” “people should be advised to buy cheaper drugs in such online pharmacies as these,” and “people should be warned against buying cheaper drugs in such online pharmacies as these.”

To assess students’ interpretations of low drug costs, they were asked to check the most plausible explanation for a lower cost of Beozine in Pharmacy B. Eight choices, such as “few regulations” or “high sales volume,” were listed and explained.

Other measured variables were demographics (gender and age) and education (health major, yes or no, and the number of college credits earned to date). Self-reported health was measured using an eVAS where 0 = very poor and 10 = excellent. An Internet-related belief, “The quality of health information found through Web search engines, such as Google or Yahoo, is usually higher than health information in libraries,” was also measured with an eVAS with end points marked 0 = strongly disagree and 10 = strongly agree. Finally, there was a measure of health-related Internet behavior, that is, whether an individual had used information from general Internet searches for health decision making for themselves or to help others.

## Results

### Study Participants

Between September 2005 and March 2008, 2096 students completed the RRSA as an optional educational activity. The participation rate was 78%.The participants were drawn from the population of undergraduate and graduate students enrolled in health-related courses offered by a Midwestern university. Although the study participants came from a variety of graduate and undergraduate programs in health-related sciences, the vast majority of students (75%) were enrolled in an undergraduate healthy lifestyles course. Data from 1914 study participants who took more than 15 minutes to complete the RRSA were used for analyses; 172 records (less than 1%) were excluded due to a short time taken to complete the assessment. Approximately 73% of students were female, 77% were younger than 22 years old, and 44% had selected a health-related field of study as their main academic concentration. Most students (90%) were completing a four-year undergraduate degree, the remainder had earned their bachelor’s or master’s degrees.

### Evaluation of Rogue Pharmacies

**Figure 1:**
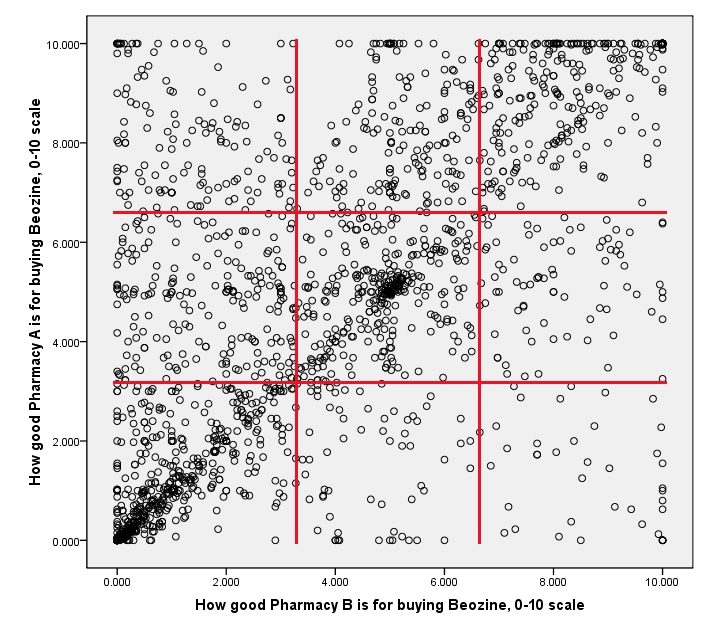
Scatter plot of respondents’ ratings of Pharmacies A and B (n = 1914)

In Figure 1, each of the 1914 respondents is designated as a dot, the placement of which is based on how this respondent rated Pharmacy A and Pharmacy B. There was a lot of variation in how the respondents rated pharmacies. Figuratively speaking, respondents’ ratings were “all over the map.” A visible diagonal line indicates that ratings of Pharmacy A and Pharmacy B were correlated (Pearson’s *r* = 0.61, *P* < .001, one-tailed). Students in the top right corner of the graph (15% of all respondents) thought that both pharmacies were great places to buy the drug, whereas students in the bottom left corner were more cautious in their evaluations. The top left triangle has more dots than the bottom right triangle, which means that Pharmacy A was evaluated more favorably than Pharmacy B. Indeed, the median rating for Pharmacy A was 4.95 (mean 4.72, SD 3.23) and the median for Pharmacy B was 3.55 (mean 3.82, SD 3.04).

In Figure 1, red lines divide the scatter plot into nine quadrants based on bottom one-third, middle one-third, and top one-third of the ratings (out of 10) of each pharmacy. Percent of respondents in each cell is presented in Table 2. Only 31% of respondents gave low ratings to both pharmacies.

Of interest is the conditional probability of B=b | A = a, as shown in Table 3. Respondents who rated Pharmacy A low (bottom one-third) would likely rate Pharmacy B low: Probability (B=low|A=low) = .838.

Table 4 shows distributions for study participants’ ratings of the Internet pharmacies. A relatively small number of participants (between 17% and 25%) had highly negative judgments of the two pharmacies as sources for obtaining the drug. About half of the participants (49.8%) provided a positive evaluation of A and over one-third (37.3%) of study participants rated Pharmacy B favorably, as indicated by ratings of five or higher. Students’ perceptions of Internet pharmacies varied greatly, as indicated by a wide range of responses and high standard deviations.

**Table 2:** Joint and marginal probabilities for respondents’ ratings of online pharmacies (n = 1914)

		Pharmacy B			Total
	Rating range	0 to 3.3	3.3 to 6.7	6.7 to 10	
Pharmacy A	0 to 3.3	31.0%	3.8%	2.2%	37.0%
	3.3 to 6.7	10.4%	17.5%	4.1%	32.0%
	6.7 to 10	7.4%	8.9%	14.7%	31.0%
Total		48.9%	30.1%	21.0%	100.0%

**Table 3:** Conditional probabilities for respondents’ ratings of online pharmacies (n = 1914)

		Pharmacy B			Total
	Rating Range	0 to 3.3	3.3 to 6.7	6.7 to 10	
Pharmacy A	0 to 3.3	83.8%	10.3%	5.9%	100.0%
	3.3 to 6.7	32.5%	54.6%	12.9%	100.0%
	6.7 to 10	23.9%	28.7%	47.4%	100.0%

**Table 4:** Distributions for respondents’ ratings of online pharmacies (n = 1914)

Rating^a^	Cumulative Percent of Respondents	
	Pharmacy A	Pharmacy B
0 up to 1.0	17.7	25.0
1.0 up to 2.0	25.7	35.4
2.0 up to 3.0	32.7	44.7
3.0 up to 4.0	41.0	52.4
4.0 up to 5.0	50.2	62.7
5.0 up to 6.0	63.5	73.8
6.0 up to 7.0	70.3	80.7
7.0 up to 8.0	78.3	86.8
8.0 up to 9.0	86.1	93.5
9.0 up to 10.0	100.0	100.0

^a^Ratings were made on a 0 to 10 electronic visual analog scale with a .025 increment and end points marked as “0 = Very bad” and “10 = Very good.”

Over 22% of respondents indicated that they would recommend Pharmacy A to friends and family, as compared to 10% of respondents who would recommend Pharmacy B. While 16% of respondents reported that people should be advised to buy cheaper drugs at these Internet pharmacies, the majority of respondents (62%) suggested that people should be warned against buying drugs at Pharmacy A and Pharmacy B.

Table 5 shows reasons commonly chosen by the study participants to explain why Pharmacy B sells Beozine much cheaper than a local neighborhood pharmacy. Both pharmacies offered drugs at a lower price than a neighborhood pharmacy. To keep the assessment completion time under 40 minutes, participants were asked to explain a cheaper price at only one pharmacy, which displayed a greater number of features that put into question its legitimacy. The majority of participants explained cheaper prices by a lack of regulatory standards with which the pharmacy must comply, followed by the fact that Internet pharmacies’ operational costs are lower than operational costs of traditional, neighborhood pharmacies. Other commonly chosen reasons were potentially lower quality of drugs, supplementary revenues from advertising, customer pressures (comparison shopping), higher sales volume, and supplementary revenues from selling information about customers.

**Table 5:** Respondents’ explanations for low cost of Beozine sold by Pharmacy B

Reasons		Percent of Respondents^a^
**Negative reasons**		
	Few regulations: pharmacy B may follow fewer operational guidelines or service standards than neighborhood pharmacies	60.0
	Low quality of drugs: pharmacy B may not meet the standards of drug quality that neighborhood pharmacies must meet	47.5
	Selling customer information: revenue from information sold to others may be used to lower prices in Pharmacy B	30.0
**Neutral reasons**		
	Low operation costs: it may cost less to operate Pharmacy B (eg, because customers type their own information)	56.7
	Advertising: revenue from online ads may be used to lower prices in Pharmacy B	37.1
	Comparison shopping: the customers of Pharmacy B may compare prices, demand free shipping, discounts, coupons or other incentives	34.6
	High sales volume: more people may buy drugs online than in neighborhood pharmacies, which lowers prices in Pharmacy B	30.3
	None of the above	7.5

^a^n = 1914. The sum of percentages exceeds 100% because the respondents could choose more than one reason.

To better understand these responses, reasons for low drug cost were sorted into three categories: (1) negative reasons that have the potential to cause harm to pharmacy customers, (2) neutral reasons, and (3) none of the above. When explaining low cost of Beozine at Pharmacy B, 59% of respondents checked a mix of neutral and negative reasons, 19% of respondents checked only neutral reasons, 14% of respondents checked only negative reasons, and the remaining 8% of respondents checked a “none of the above” option. The more negative reasons a respondent checked, the more likely he or she was to negatively judge Pharmacy B as a place to buy Beozine (F_3,1910_ = 66.3, *P* < .001). The number of neutral reasons checked also had a significant relationship with pharmacy ratings but in the opposite direction. Those who checked several neutral reasons for cheap prices were more likely to assign higher ratings to Pharmacy B than those who checked few or no neutral reasons (F_4,1909_ = 23.4, *P* < .001).

Next, as a proxy measure of critical judgment, a pharmacy evaluation index was calculated as a mean of five factor scores: (1) ability to recognize negative reasons for low costs of Beozine at Pharmacy B; (2) willingness to recommend Pharmacy A to friends and family; (3) willingness to recommend Pharmacy B to friends and family; (4) rating of Pharmacy A as a place to purchase Beozine; and (5) rating of Pharmacy B as a place to purchase Beozine. The index ranged from 0 to 1 where 0 was “very bad judgment” and 1 was “very good judgment.” Each factor score was scaled from 0 to 1 and, if needed, recoded so that higher scores consistently demonstrated better judgment of pharmacies. For example, factors 4 and 5—ratings of pharmacies—were originally scaled 0 to 10 where 10 meant “good place to buy the drug.” Any rating between 0 and .999 was recoded as one and any rating between 1 and 10 was recoded as zero. Scaled 0 to 1 with a rating of one representing better judgment, factors 4 and 5 were prepared for inclusion in the pharmacy evaluation index.

Independent-samples *t* tests were conducted to evaluate if individuals who use Internet information for making health decisions demonstrate better critical judgment skills, as indicated by the pharmacy evaluation index. The results were counter to expectations. Study participants who made health decisions using information they found by searching Google or another Internet search engine (n = 762) had a lower mean (SD) score on a pharmacy evaluation index than individuals who did not make such decisions (n = 1,152): 0.61 (0.23) versus 0.65 (0.21). Similarly, individuals who helped another person (eg, a relative or a friend) to make a health decision based on the information they located in Google or another Internet search engine (n = 604) had a lower mean (SD) judgment score of online pharmacies than individuals who did not help others to make such decisions (n = 1310): 0.61 (0.24) versus 0.65 (0.21). Both *t*tests were significant, *t*_1912_ = 3.62, *P* < .001 and t_1912_ = 3.75, *P* < .001, respectively. The effect size was small; Cohen’s d was .18 for both comparisons.

Predictors of the pharmacy evaluation index were examined using a hierarchical regression analysis. The predictors were demographics (gender and age), education (health major, yes or no, and the number of college credits earned to date), self-reported health, Internet-related beliefs (“The quality of health information found through Web search engines, such as Google or Yahoo, is usually higher than health information in libraries”) and Internet behaviors (applying health information found by searching general search engines to health decisions). As can be seen in Table 6, Model 1 took into account demographics, education, and self-reported health. Nearly 5% of the variance in the dependent variable was accounted for by education credits, age, health major, and self-reported health. All of these variables, except self-reported health, were significantly and positively related to the pharmacy evaluation index. Gender was not a significant predictor of pharmacy judgment. Model 2 included the same predictors as Model 1 plus Internet-related beliefs and behaviors. It accounted for a significant yet small (8%) amount of the variance in the pharmacy evaluation index. After controlling for Model 1 predictors, whether an individual used information from general Internet searches for health decision making (for self or to help others) was a significant negative predictor, as well as a belief in the high quality of Internet health information. Together, these variables explained 3% of additional variance in the pharmacy evaluation index. The practical significance of this finding is limited by a small effect size.

**Table 6:** Summary of hierarchical regression analysis for variables predicting a pharmacy evaluation index (n = 1914)

	Model 1			Model 2		
Variable	B^a^	SE^b^B	Beta^c^	B^a^	SE^b^B	Beta^c^
Age	0.01	0.00	0.09^d^	0.01	0.00	0.10^d^
Gender	0.02	0.01	0.03	0.01	0.01	0.02
College credits earned	0.04	0.01	0.09^d^	0.03	0.01	0.07^d^
Health major	0.02	0.00	0.13^d^	0.01	0.00	0.10^d^
Self-reported health	-0.01	0.00	-0.08^d^	-0.01	0.00	-0.08^d^
Belief in the high quality of Internet health information				-0.03	0.01	-0.06^d^
Made health decisions^e^				-0.02	0.00	-0.17^d^
R^2^		.05			.08	
F change for R^2^		19.56^d^			34.66^d^	

^a^Unstandardized regression coefficient (uses units unique to each variable)

^b^Standard error of B

^c^Standardized regression coefficient (uses the same units for all variables in the equation)

^d^Significant at the .01 level

^e^Whether an individual used information from general Internet searches for health decision making, for self, or to help others

## Discussion

The findings of this study indicate that university students are not making appropriate judgments about health information that is provided on the Internet. The two Internet pharmacies used in this study had multiple untrustworthy features that were borrowed from five actual pharmacy websites that the authors considered to be potentially dangerous to consumers. Yet, almost one-half of the study population rated the Pharmacy A site favorably, while over one-third rated Pharmacy B in a favorable manner. It is interesting to note that some of the participants who gave these rogue pharmacies positive evaluations would not recommend them to family and friends. In fact, 62% of the study population would warn family and friends against using them. Even so, about one quarter of respondents would recommend Pharmacy A to friends and family. An alarming number of college-enrolled respondents (16%) thought that people should be advised to buy cheaper drugs at such Internet pharmacies.

When asked about why Beozine was cheaper at an Internet pharmacy versus the local pharmacy, the respondents checked several explanations. First, 60% of respondents believed that cheaper drugs were due to less regulatory restrictions, as compared to local pharmacies. Perhaps these respondents noticed that Pharmacies A and B were located outside of the US and took this as an indicator, perhaps in conjunction with other untrustworthy features, that these pharmacies might not be compliant with the US laws. An alternative explanation would be that the respondents did not believe that Internet pharmacies could be regulated as well as storefront pharmacies. Future research should continue to monitor the level of consumer awareness of pharmacy standards and accreditation. Do consumers know that all US Internet pharmacies must comply with the same regulations and face the same penalties for non-compliance as storefront pharmacies or clinics [10]? Do they know to look for a Verified Internet Pharmacy Practice Sites (VIPPS) logo that indicates that the pharmacy was accredited by the National Association of Boards of Pharmacy?

Only 30% of the respondents stated the lower drug costs might be due to the Internet pharmacies selling their information to other companies, despite the fact that both pharmacies asked for large amounts of personal customer information. In addition to these information requests, a large number of other features communicated potential danger, such as misleading statements, suspicious disclaimers, unsupported claims, requests for personal information, typographical errors, and no way to contact a live person by phone. But these plentiful signs of danger, absence of credibility markers, and very low drug prices did not arouse consumer suspicion in at least one-third of young people who participated in this study.

Individuals who linked low drug costs to signs of danger (few regulations, low quality of drugs and selling customer information) had more negative evaluations of the Internet pharmacies than those who cited neutral reasons. The actual rogue pharmacy websites we accessed offered their customers multiple neutral reasons, saying that their low prices were a result of high sales volumes, low operation costs, and consumer pressure due to comparison shopping. About 30% of study participants thought that the drugs could be cheaper online due to volume sold. As warned by Palumbo [13], increased sales volumes may not result in lowered drug costs but may result in more counterfeit drugs in the future.

We also examined if those who used Internet information to make health decisions had better judgment skills. It was not the case. In fact, individuals who used general search engines had worse evaluation skills than students who reported more traditional methods for making health decisions. Additionally, it was found that those helping others make informed health decisions using the Internet information had worse judgment than those who did not. In other words, people with worse judgment (controlling for all other variables) are the ones most likely to use information to help others. Perhaps these individuals are more eager to use any information versus quality information. Not very skilled in evaluating the Internet pharmacies, these individuals may then recommend buying drugs to others. This was an interesting finding that was not hypothesized a priori and had a small effect size.

In this study, the evaluation of Internet information was positively correlated with students’ age, number of earned college credits, and a health-related major. Therefore, it would be expected that older individuals with more college education should be able to make better judgments about the health information provided online. As compared with younger people, older consumers of information would have had more experience with a wide variety of media—interpersonal communication, TV, radio, print, etc—and might have learned to be cautious. Their folk wisdom that people should not believe everything they see, hear, or read may transfer from old media to new media, even for those with limited Internet experience. It is also likely that any higher education, and especially education in health sciences, serves to improve electronic health literacy skills, such as the skills involved in determining the credibility of health websites. On the other hand, individuals with low literacy and those with less formal education are expected to be susceptible to making a purchase from a rogue Internet pharmacy.

Motivated by high profits from illegal drug sales, creators of rogue Internet pharmacies are likely to employ new, sophisticated ways to lure consumers to their products. For example, when the popular press was covering the price advantage of Canadian pharmacies, a large number of Internet pharmacies, including those not based in Canada, exploited the opportunity to gain consumer trust by presenting themselves as Canadian pharmacies [32]. From this study, it can be determined that many college-educated young people cannot see the signs of danger displayed by rogue Internet pharmacies, and those that have skills and competencies may not use them when viewing Internet pharmacy information. An even greater number of individuals are likely to be misled by seller websites that show fewer signs of untrustworthiness and greater surface credibility—marked by professional Web design, a polished appearance, or attractive graphics—than the websites used in this study.

Suggestions for future study include designing research that can directly test the relationships noted here to find out if these results can be replicated in other settings and populations. Since these findings were not predicted or hypothesized, but found in post-hoc analyses, additional research is warranted to purposefully test these relationships. The RRSA had only a few pharmacy questions that explored a limited number of issues in the population of college students. A more sophisticated design could help to explain some of the presented findings. Using this as a preliminary study, it can be stated that better educated consumers have higher electronic information literacy and better health-related decision making. Another interesting direction for future study is to examine the relationship between consumer attitudes about the enforcement of intellectual property laws and their willingness to buy from rogue Internet pharmacies. How many consumers see small online sellers as a viable alternative to traditional drug distribution channels? Do online shoppers believe that traditional drug distribution channels are tightly controlled by large drug companies that overprice their patented drugs?

It is suggested that a two-tiered approach be utilized for consumers that would include both educational programs and regulatory efforts. Health care professionals, including health educators, need to develop consumer education programs and communication campaigns that explain the variable quality of Web-based health information and that build information evaluation skills and otherwise promote digital media literacy. This study highlighted the importance of making consumers aware of the concerns with medications purchased online and with Internet pharmacies and the importance of explaining to consumers the reasons for very low drug costs and the dangers of self-diagnosis. Further, consumer education is needed about the medications themselves, as Internet pharmacies are often not providing adequate information or education [12]. Because health educators and consumer educators have relatively easy access to young Internet users, these users can be included in pilot tests of new programs developed to educate these individuals about illegitimate pharmacies and to build their health information literacy skills.

From a governmental perspective, the federal system cannot lose sight of the dangers of Internet pharmacies. Although much progress has been made in regard to regulating US Internet pharmacies, there is still much work to be done in regulating foreign pharmacies and curbing the danger they pose to consumers. With improved regulation, international collaboration, and consumer education, there will be an increased assurance of safety for those wishing to utilize Internet pharmacies.

In sum, our findings suggest that at least a quarter of consumers would consider using rogue sellers of medications similar to the ones we used in this study. Many more consumers are likely to be misled by rogue Internet pharmacies that (1) use website designs that appear more professional, (2) better veil their untrustworthy features, and (3) mimic reputable websites to a greater extent than the Internet pharmacies used in this study.

## Abbreviations

eVAS: electronic visual analog scale
FDA: The US Food and Drug Administration
RRSA: Research Readiness Self-Assessment
VIPPS: Verified Internet Pharmacy Practice Sites

## Multimedia Appendix 1

Website of Pharmacy A and Website of Pharmacy B

## Multimedia Appendix 2

Video (different formats) that demonstrates interactive web-based questions about Pharmacies A and B and navigation of their websites
