# Supplementary material for: Dirt Cheap and Without Prescription: How Susceptible are Young US Consumers to Purchasing Drugs From Rogue Internet Pharmacies?
Source: J Med Internet Res. 2010 Apr 26;12(2):e11. doi: 10.2196/jmir.1520 (PMC2885783; doi:10.2196/jmir.1520)
Supplement: Supplementary file 1 [file jmir_v12i2e11_app1.pdf]

DIRT CHEAP AND WITHOUT PRESCRIPTION:

How susceptible are young U.S. consumers to purchasing drugs from rogue internet pharmacies?

By Lana Ivanitskaya, Jodi Brookins-Fisher, Irene O'Boyle, Danielle Vibbert, Dmitry Erofeev and Lawrence Fulton

Journal of Medical Internet Research

Corresponding author:
Lana Ivanitskaya, Ph.D.
ivani1sv@cmich.edu
Central Michigan University

Questions about pharmacies come from *Research Readiness Self-Assessment*, eHealth version for students

Research Readiness Self-Assessment
Central Michigan University

Main Menu
Home
Contact us
Credits

"How To" of the Information Age - Fast. Smart. Safe.

Self-Assessment of eHealth Competencies for Students

Lana Ivanitskaya, Ph.D.
Version RRSA-CMU-h-2. Updated: Oct 30, 2009

Why assess my skills?

When you research health topics, you want to get information that is accurate, balanced, and complete. It's not easy to find that. Here is why,

- Of 228 TV health segments reviewed by health information experts, "many of the stories [were] bad and they [weren't] getting much better" (1).
- Medical science reports in newspapers and magazines are sometimes distorted in the direction of sensation (2).
- It's cheap and fast to publish online. A person can create a website that looks as sleek as a website by a respected organization. There are no clear standards for internet publishing. It's hard to tell apart health news and ads. Copied content is not updated (3). Finally, some of the best health information is online but a password is needed to see it. You may be able to access it as a student...if you know what to do.

Get the picture? It may be easy to get health information but sorting through it gets tricky. It matters what source you use, how you search, what results you pick and how you use what you find. This tool helps you be better and faster at finding what you need online.

Website of Pharmacy A

Note: For the purposes of this demonstration the top-level domain was replaced with "xxx."

PharmacyBest.###

Home | About Us | Contact | Search

About Our Services

ONLINE CONSULTATIONS

Online consultations are the latest concept in health care utilizing the Internet to improve patient access to physician care. A very large percentage of United States physicians currently pescribe medications by reviewing a client's medical history without an in-person physical exam. The patient completes an online questionnaire in the same manner as they would in a physician's office.

FREE MEDICAL REVIEW

If you do not have a prescription from your physician or doctor, most of the online pharmacies provide online consultations some of which are complimentary FREE and others charge a fee for this type of service. We offer FREE medical review with prescription from real doctor. First of all, you open up an account, by submitting your name, address, phone number, credit card number and insurance information if applicable.

HIGH QUALITY

Home
Search
Contact
Links

NO PERSCRIPTION REQUIRED!

Our staff can prescribe medications based on a detailed questionnaire. We would review the information you submit and respond within **one hour!**

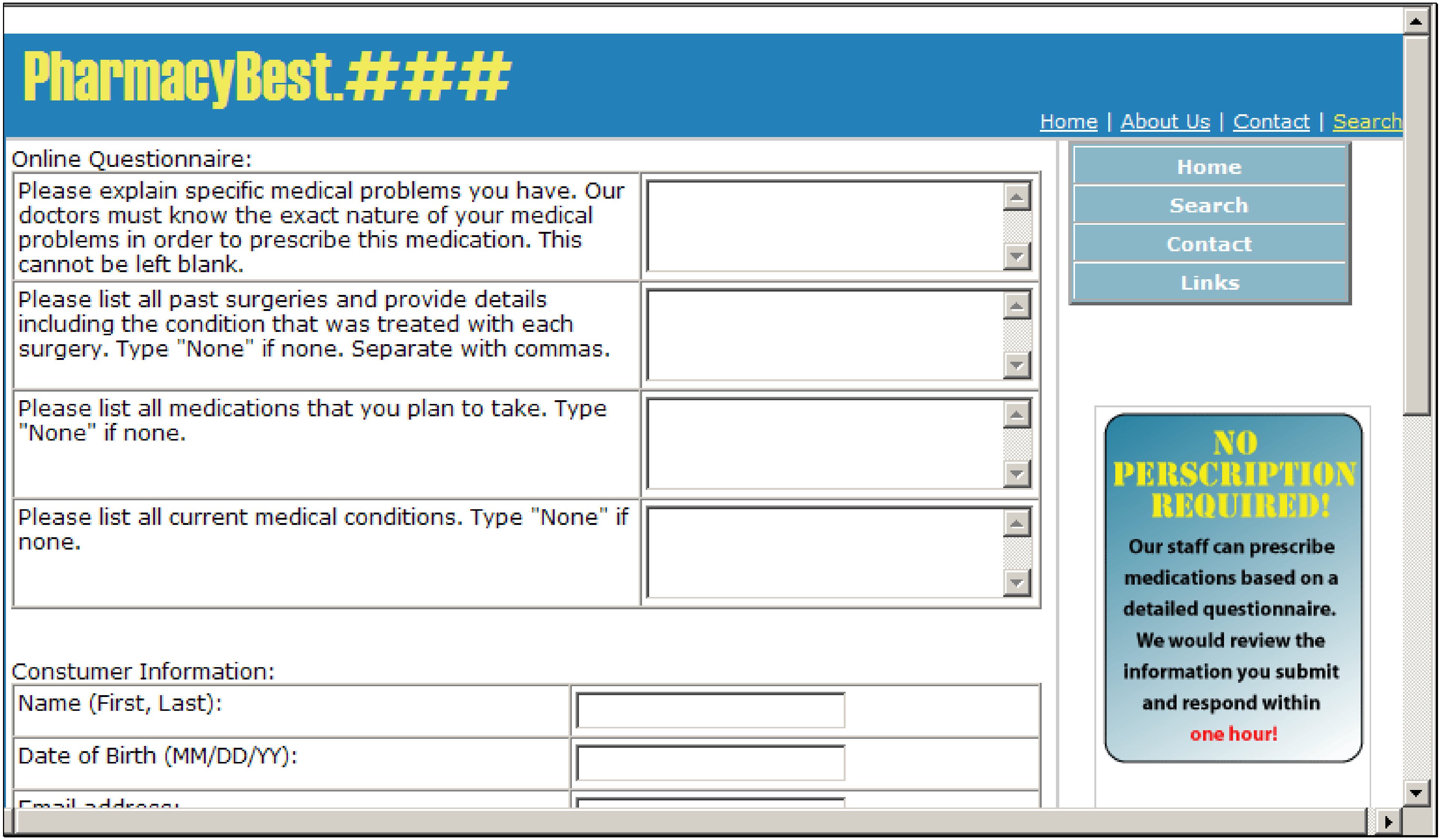

PharmacyBest.###

Home | About Us | Contact | Search

Online Questionnaire:

Please explain specific medical problems you have. Our doctors must know the exact nature of your medical problems in order to prescribe this medication. This cannot be left blank.	
Please list all past surgeries and provide details including the condition that was treated with each surgery. Type "None" if none. Separate with commas.	
Please list all medications that you plan to take. Type "None" if none.	
Please list all current medical conditions. Type "None" if none.	

Constumer Information:

Name (First, Last):	
Date of Birth (MM/DD/YY):	

Home
Search
Contact
Links

NO PERSCRIPTION REQUIRED!

Our staff can prescribe medications based on a detailed questionnaire. We would review the information you submit and respond within one hour!

PharmacyBest.###

Home | About Us | Contact | Search

Search « Beozine

Found 1 result(s) for **Beozine at US $37.99!**

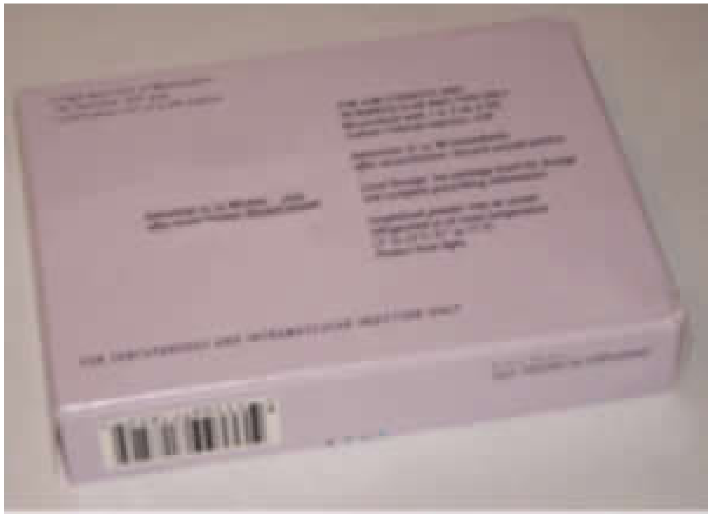

- Drug Name: Beozine
- Price: **US $37.99**
- Availability: IN STOCK!!
- About: Now also available as a gel!
- **More Info**

BUY NOW!

- Home
- Search
- Contact
- Links

NO PERSCRIPTION REQUIRED!

Our staff can prescribe medications based on a detailed questionnaire. We would review the information you submit and respond within one hour!

ABOUT OUR SERVICES

Website of Pharmacy B

Note: For the purposes of this demonstration the top-level domain was replaced with "xxx."

DrugsForLess [dot]

Contact Us

Navigation

Home

Contact

Search

FAQ

Drugs For Less [dot]

No need to meet your doctor if your prescription expired, we can update your prescription. We offer Wholesale Pricing, Prescription Updates, Worldwide Shipping, Private Online Ordering, and Discreet Packaging.

Today's Special!

Beozine retails for US$200, we sell for $59.50!

Customers□ Comments

"Thank you for being my online doctor! I have been getting your medications for the past month. I had had 2 major surjeries, both with complications, in the past year and just never started feeling "well" again. I did some investigation, and realized I probably had an allergy, and your doctors confirmed it. I don't know how they could tell it right away, but I am thankful that I once again have energy and feel "well" once again. Thanks,"

DrugsForLess.###
Contact | Disclaimer | Home

Your transaction will be processed in USD dollars through our corporation at Banco del Valle, Calle Fuentos Arias, Piso No. 43, Apartado 7364, Panama 11, R. de Panama.

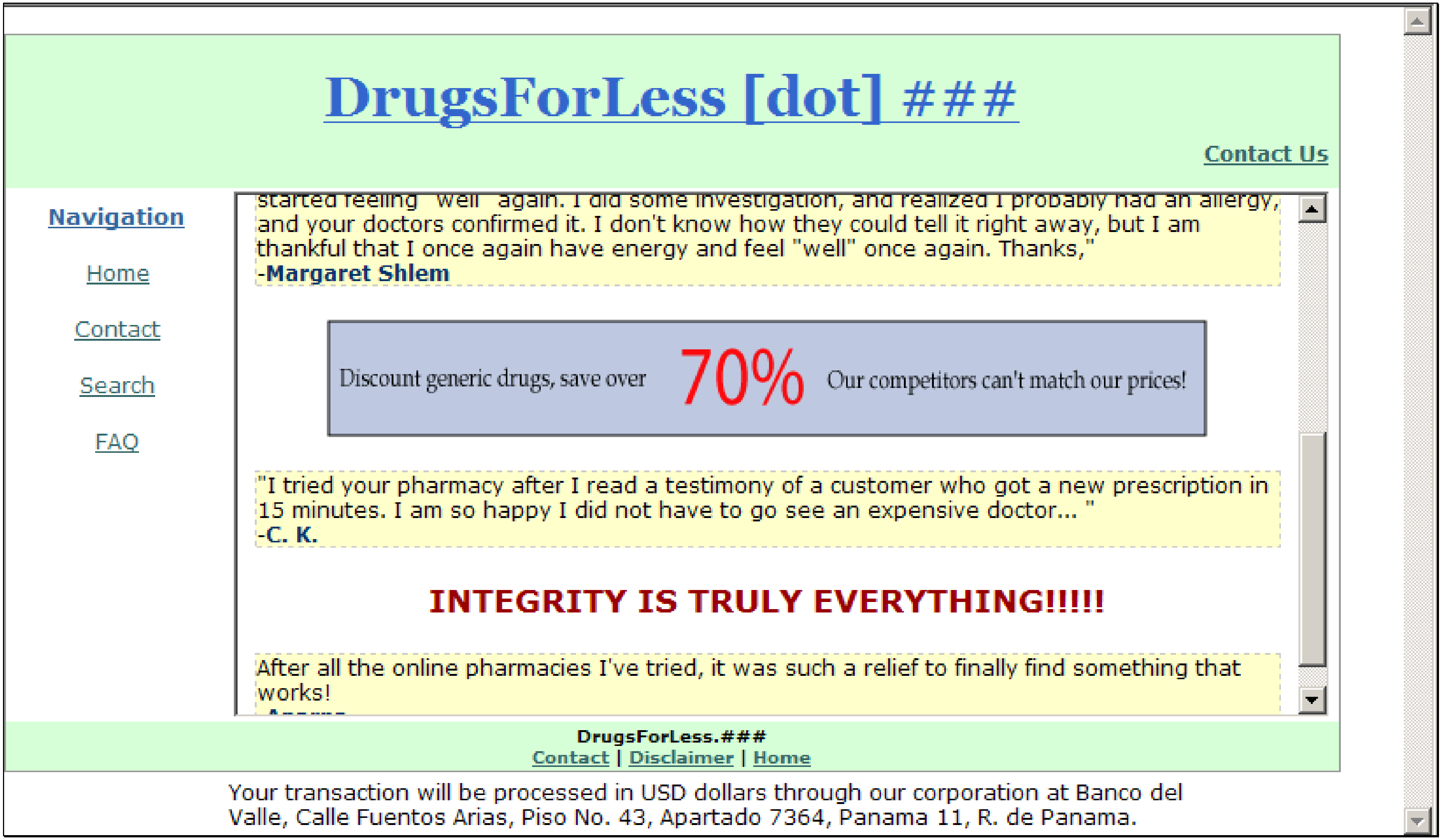
DrugsForLess [dot] ###
Contact Us
Navigation
Home
Contact
Search
FAQ
started feeling "well" again. I did some investigation, and realized I probably had an allergy, and your doctors confirmed it. I don't know how they could tell it right away, but I am thankful that I once again have energy and feel "well" once again. Thanks,"
-Margaret Shlem
Discount generic drugs, save over
70%
Our competitors can't match our prices!
"I tried your pharmacy after I read a testimony of a customer who got a new prescription in 15 minutes. I am so happy I did not have to go see an expensive doctor... "
-C. K.
INTEGRITY IS TRULY EVERYTHING!!!!!
After all the online pharmacies I've tried, it was such a relief to finally find something that works!
DrugsForLess.###
Contact | Disclaimer | Home
Your transaction will be processed in USD dollars through our corporation at Banco del Valle, Calle Fuentos Arias, Piso No. 43, Apartado 7364, Panama 11, R. de Panama.

DrugsForLess [dot]

Contact Us

Navigation

Home

Contact

Search

FAQ

Search « Beozine

Found 1 result(s) for **Beozine at US $37.99!**

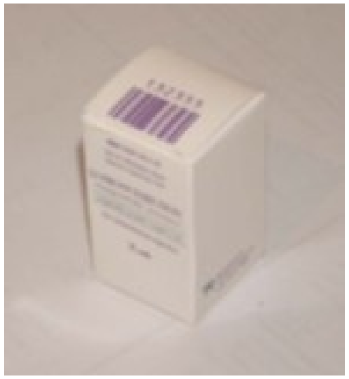

- Drug Name: Beozine
- Price: **US $59.50**
- Availability: IN STOCK!!
- About: Generic Drug
- **More Info**

BUY NOW!

buynow

DrugsForLess.###
Contact | Disclaimer | Home

Your transaction will be processed in USD dollars through our corporation at Banco del Valle, Calle Fuentos Arias, Piso No. 43, Apartado 7364, Panama 11, R. de Panama.
